# Supplementary material for: Effectiveness of health voucher scheme and micro-health insurance scheme to support the poor and extreme poor in selected urban areas of Bangladesh: An assessment using a mixed-method approach
Source: PLoS One. 2021 Nov 1;16(11):e0256067. doi: 10.1371/journal.pone.0256067 (PMC8559931; doi:10.1371/journal.pone.0256067)
Supplement: S1 Table — (DOCX) [file pone.0256067.s001.docx]

S1 Table. Out-of-pocket payments (Euro) of scheme enrolees in the last 6 months period (without outliers)

| **Items** | **HVS** | | | | | | | | |  | **MHI Dhaka** | | | |
| --- | --- | --- | --- | --- | --- | --- | --- | --- | --- | --- | --- | --- | --- | --- |
|  | **Dhaka** | | | |  | **Chattogram** | | | |  |  |  |  |  |
|  | **N** | **Mean** | **Median** | **SE(Mean)** |  | **N** | **Mean** | **Median** | **SE(Mean)** |  | **N** | **Mean** | **Median** | **SE(Mean)** |
| Consultation fee | 296 | 3.1 | 2.1 | 0.2 |  | 69 | 3.0 | 2.1 | 0.4 |  | 120 | 3.9 | 3.2 | 0.4 |
| Medicine cost | 840 | 7.8 | 4.2 | 0.4 |  | 116 | 9.8 | 5.3 | 1.0 |  | 314 | 9.0 | 5.3 | 0.6 |
| Bed charges | 14 | 11.1 | 5.3 | 4.0 |  | 1 | 0.1 | 0.1 | - |  | 11 | 7.3 | 2.6 | 3.4 |
| Diagnostic cost | 164 | 13.7 | 9.8 | 1.0 |  | 38 | 8.8 | 6.3 | 1.1 |  | 79 | 13.1 | 10.6 | 1.1 |
| Transport cost | 409 | 1.6 | 0.8 | 0.1 |  | 255 | 1.3 | 0.6 | 0.1 |  | 201 | 1.9 | 1.1 | 0.2 |
| Tips | 44 | 2.5 | 2.1 | 0.4 |  | 5 | 2.1 | 1.1 | 0.9 |  | 25 | 2.3 | 2.1 | 0.3 |
| Caregiver cost | 8 | 4.4 | 2.6 | 1.4 |  | 13 | 4.2 | 3.2 | 1.1 |  | 28 | 2.8 | 2.1 | 0.4 |
| Other cost | 43 | 6.9 | 2.6 | 1.4 |  | 27 | 3.9 | 2.1 | 0.9 |  | 35 | 2.0 | 1.6 | 0.3 |
| **Total** | **1,005** | **10.9** | **4.2** | **0.5** |  | **311** | **7.1** | **1.1** | **0.8** |  | **362** | **13.8** | **6.3** | **0.9** |
