# Supplementary material for: Effectiveness of health voucher scheme and micro-health insurance scheme to support the poor and extreme poor in selected urban areas of Bangladesh: An assessment using a mixed-method approach
Source: PLoS One. 2021 Nov 1;16(11):e0256067. doi: 10.1371/journal.pone.0256067 (PMC8559931; doi:10.1371/journal.pone.0256067)
Supplement: S2 Table — (DOCX) [file pone.0256067.s002.docx]

S2 Table. Out-of-pocket healthcare payments in Euro of scheme beneficiaries in the last 6 months period (including outliers)

| **Items** | **HVS** | | | | | | | | |  | **MHI** | | | |
| --- | --- | --- | --- | --- | --- | --- | --- | --- | --- | --- | --- | --- | --- | --- |
|  | **Dhaka** | | | |  | **Chattogram** | | | |  | **Dhaka** | | | |
|  | **N** | **Mean** | **Median** | **SE (Mean)** |  | **N** | **Mean** | **Median** | **SE (Mean)** |  | **N** | **Mean** | **Median** | **SE (Mean)** |
| Consultation fee | 363 | 4.7 | 2.1 | 0.4 |  | 83 | 4.2 | 2.1 | 0.6 |  | 153 | 6.2 | 3.2 | 0.9 |
| Medicine cost | 933 | 16.6 | 5.3 | 1.7 |  | 134 | 15.3 | 8.5 | 1.7 |  | 357 | 13.5 | 6.3 | 1.0 |
| Bed charges | 59 | 33.4 | 15.8 | 5.2 |  | 8 | 29.9 | 21.7 | 9.7 |  | 34 | 24.2 | 15.3 | 6.0 |
| Diagnostic cost | 244 | 25.4 | 15.3 | 2.5 |  | 56 | 16.8 | 10.6 | 2.5 |  | 118 | 22.5 | 15.8 | 2.5 |
| Transport cost | 500 | 2.7 | 1.1 | 0.2 |  | 272 | 1.9 | 0.6 | 0.2 |  | 244 | 3.1 | 1.1 | 0.3 |
| Tips | 54 | 2.8 | 2.6 | 0.3 |  | 8 | 3.6 | 2.6 | 1.2 |  | 35 | 3.0 | 2.6 | 0.4 |
| Caregiver cost | 34 | 10.0 | 8.5 | 1.7 |  | 26 | 7.0 | 5.3 | 1.1 |  | 59 | 6.2 | 4.2 | 0.8 |
| Other cost | 74 | 24.3 | 5.8 | 6.9 |  | 39 | 5.2 | 4.2 | 0.8 |  | 65 | 23.7 | 2.6 | 16.2 |
| **Total** | **1,105** | **26.2** | **5.3** | **2.5** |  | **329** | **13.7** | **1.1** | **1.9** |  | **406** | **29.6** | **7.8** | **3.5** |
