# Supplementary material for: Effectiveness of health voucher scheme and micro-health insurance scheme to support the poor and extreme poor in selected urban areas of Bangladesh: An assessment using a mixed-method approach
Source: PLoS One. 2021 Nov 1;16(11):e0256067. doi: 10.1371/journal.pone.0256067 (PMC8559931; doi:10.1371/journal.pone.0256067)
Supplement: S3 Table — (DOCX) [file pone.0256067.s003.docx]

S3 Table. Out-of-pocket payments (Euro) for Maternal, Newborn and Child Health (MNCH) services (per service) (without outliers)

| **Types of MNCH services** | **HVS** | | | | | | | | |  | **MHI** | | | |  |
| --- | --- | --- | --- | --- | --- | --- | --- | --- | --- | --- | --- | --- | --- | --- | --- |
|  | **Dhaka** | | | |  | **Chattogram** | | | |  | **Dhaka** | | | |  |
|  | **N** | **Mean** | **Median** | **SE (Mean)** |  | **N** | **Mean** | **Median** | **SE (Mean)** |  | **N** | **Mean** | **Median** | **SE (Mean)** | |
| ANC | 90 | 6.1 | 3.2 | 1.0 |  | 14 | 1.1 | 1.1 | 0.1 |  | 28 | 6.7 | 5.1 | 1.2 | |
| Normal delivery | 75 | 16.2 | 13.3 | 1.7 |  | 9 | 8.9 | 3.2 | 5.1 |  | 49 | 18.4 | 8.5 | 2.9 | |
| C-section delivery | 28 | 37.7 | 37.0 | 4.1 |  | 1 | 47.5 | 47.5 | - |  | - | - | - | - | |
| PNC | 5 | 12.4 | 10.6 | 4.4 |  | 1 | 1.1 | 1.1 | - |  | 9 | 14.1 | 1.3 | 8.0 | |
