# Supplementary material for: Effectiveness of health voucher scheme and micro-health insurance scheme to support the poor and extreme poor in selected urban areas of Bangladesh: An assessment using a mixed-method approach
Source: PLoS One. 2021 Nov 1;16(11):e0256067. doi: 10.1371/journal.pone.0256067 (PMC8559931; doi:10.1371/journal.pone.0256067)
Supplement: S4 Table — (DOCX) [file pone.0256067.s004.docx]

S4 Table. Out-of-pocket payments in Euro for Maternal, Newborn and Child Health (MNCH) services (per service) including outliers

| **Types of MNCH services** | **HVS** | | | | | | | | |  | **MHI** | | | |  |
| --- | --- | --- | --- | --- | --- | --- | --- | --- | --- | --- | --- | --- | --- | --- | --- |
|  | **Dhaka** | | | |  | **Chattogram** | | | |  | **Dhaka** | | | |  |
|  | **N** | **Mean** | **Median** | **SE (Mean)** |  | **N** | **Mean** | **Median** | **SE (Mean)** |  | **N** | **Mean** | **Median** | **SE (Mean)** | |
| ANC | 93 | 12.1 | 3.7 | 3.9 |  | 14 | 1.1 | 1.1 | 0.1 |  | 29 | 9.7 | 5.3 | 3.2 | |
| Normal delivery | 77 | 23.8 | 13.3 | 5.8 |  | 9 | 8.9 | 3.2 | 5.1 |  | 50 | 20.3 | 8.8 | 3.4 | |
| C-section delivery | 58 | 112.3 | 97.7 | 17.8 |  | 5 | 151.1 | 116.2 | 46.6 |  | 19 | 212.8 | 200.8 | 20.8 | |
| PNC | 5 | 12.4 | 10.6 | 4.4 |  | 1 | 1.1 | 1.1 | - |  | 10 | 29.6 | 1.3 | 17.1 | |
