# Supplementary material for: Effectiveness of health voucher scheme and micro-health insurance scheme to support the poor and extreme poor in selected urban areas of Bangladesh: An assessment using a mixed-method approach
Source: PLoS One. 2021 Nov 1;16(11):e0256067. doi: 10.1371/journal.pone.0256067 (PMC8559931; doi:10.1371/journal.pone.0256067)
Supplement: S5 Table — (DOCX) [file pone.0256067.s005.docx]

S5 Table. Factors associated with the utilization of medically trained providers

| **Explanatory variables** | **Utilization of MTP**  **Odds ratio (95% CI)** |
| --- | --- |
| **Type of scheme** |  |
| HVS | 1 |
| MHI | 0.677*** (0.516,0.889) |
| **Study area** |  |
| Chattogram | 1 |
| Dhaka | 0.222*** (0.157,0.314) |
| **Age group** |  |
| < 20 years | 1 |
| 20-30 years | 1.124 (0.634,1.991) |
| 30-40 years | 1.163 (0.617,2.194) |
| 40+ years | 0.994 (0.515,1.917) |
| **Sex** |  |
| Female | 1 |
| Male | 0.880 (0.684,1.133) |
| **Marital status** |  |
| Married | 1 |
| Unmarried | 1.274 (0.674,2.406) |
| Others (Widowed, Divorced and Separated) | 1.463 (0.797,2.686) |
| **Occupation** |  |
| Labour | 1 |
| Factory worker | 0.519* (0.245,1.098) |
| Rickshaw puller | 0.789 (0.380,1.640) |
| Driver | 0.475 (0.195,1.155) |
| Small business | 1.110 (0.554,2.222) |
| Service holder | 0.540* (0.288,1.013) |
| Student | 1.061 (0.546,2.060) |
| Unemployed | 1.015 (0.526,1.959) |
| Housewife | 1.518 (0.919,2.508) |
| Other | 1.467 (0.755,2.851) |
| **Household size** |  |
| 3 persons or less | 1 |
| 4-5 persons | 0.913 (0.705,1.182) |
| 6 persons or more | 1.028 (0.721,1.466) |
| **Years of schooling group** | |
| No formal education | 1 |
| Up to primary | 0.799 (0.483,1.320) |
| Secondary | 0.847 (0.511,1.404) |
| Higher secondary and above | 0.978 (0.568,1.684) |
| **Disability** |  |
| `No | 1 |
| Yes | 0.785 (0.302,2.044) |
| **Membership in NGO/cooperatives** | |
| No | 1 |
| Yes | 1.551 (0.805,2.985) |
| **Self-reported illness/service** |  |
| MNCH | 1 |
| Communicable disease | 0.0412*** (0.0222,0.0763] |
| Non-communicable disease | 0.125*** (0.0573,0.274) |
| Other condition | 0.148*** (0.0786,0.280) |
| **Assets quintiles** |  |
| Poorest | 1 |
| 2nd | 0.721* (0.505,1.031) |
| 3rd | 0.701** (0.492,0.997) |
| 4th | 0.842 (0.587,1.207) |
| Richest | 0.680** (0.474,0.976) |
| **Constant** | 175.3*** (60.62,507.0) |
| **Observations** | 2,391 |
| Log likelihood | -1,058 |
| Chi-square | 534 |
| Degrees of freedom | 31 |
| P-value | 0.000 |
| R-square | 0.202 |
