# Supplementary material for: Effectiveness of health voucher scheme and micro-health insurance scheme to support the poor and extreme poor in selected urban areas of Bangladesh: An assessment using a mixed-method approach
Source: PLoS One. 2021 Nov 1;16(11):e0256067. doi: 10.1371/journal.pone.0256067 (PMC8559931; doi:10.1371/journal.pone.0256067)
Supplement: S6 Table — (DOCX) [file pone.0256067.s006.docx]

S6 Table. Factors associated with the log of out-of-pocket healthcare expenditure

| **Explanatory variables** | **Log of OOP expenditure** |
| --- | --- |
|  | **Coeff. (95% CI)** |
| **Type of scheme** |  |
| HVS | 1 |
| MHI | 0.413*** (0.217,0.608) |
| **Study area** |  |
| Chattogram | 1 |
| Dhaka | 1.136*** (0.909,1.363) |
| **Age group** |  |
| < 20 years | 1 |
| 20-30 years | 0.314* (-0.0211,0.649) |
| 30-40 years | 0.533*** (0.140,0.925) |
| 40+ years | 0.842*** (0.410,1.275) |
| **Sex** |  |
| Female | 1 |
| Male | 0.554*** (0.350,0.757) |
| **Marital status** |  |
| Married | 1 |
| Unmarried | 0.357 (-0.0986,0.812) |
| Others (Widowed, Divorced and Separated) | 0.0230 (-0.393,0.439) |
| **Occupation** |  |
| Labour | 1 |
| Factory worker | 0.0824 (-0.489,0.654) |
| Rickshaw puller | 0.312 (-0.289,0.914) |
| Driver | -0.226 (-0.933,0.480) |
| Small business | -0.182 (-0.717,0.353) |
| Service holder | 0.267 (-0.212,0.747) |
| Student | 0.0837 (-0.434,0.601) |
| Unemployed | 0.161 (-0.342,0.663) |
| Housewife | 0.191 (-0.190,0.572) |
| Other | -0.0268 (-0.509,0.455) |
| **Household size** |  |
| 3 persons or less | 1 |
| 4-5 persons | -0.231** (-0.422,-0.0392] |
| 6 persons or more | -0.0910 (-0.348,0.166) |
| **Years of schooling group** | |
| No formal education | 1 |
| Up to primary | -0.161 (-0.549,0.227) |
| Secondary | -0.270 (-0.660,0.119) |
| Higher secondary and above | -0.169 (-0.575,0.236) |
| **Disability** |  |
| `No | 1 |
| Yes | 0.489 (-0.240,1.218) |
| **Membership in NGO/cooperatives** | |
| No | 1 |
| Yes | 0.437** (0.0434,0.830) |
| **Self-reported illness/service** | |
| MNCH | 1 |
| Communicable disease | -0.981*** (-1.271,-0.691] |
| Non-communicable disease | 0.469** (0.0520,0.886) |
| Other condition | -0.201 (-0.490,0.0869) |
| **Assets quintiles** |  |
| Poorest | 1 |
| 2nd | 0.159 (-0.104,0.422) |
| 3rd | 0.326** (0.0711,0.581) |
| 4th | 0.485*** (0.228,0.742) |
| Richest | 0.569*** (0.310,0.828) |
| **Constant** | 4.546*** (3.851,5.241) |
| **Observations** | 1814 |
| Log likelihood | -3480.4 |
| Degrees of freedom | 32 |
| P-value | 0.000 |
| R-square | 0.223 |
