# Supplementary material for: Effectiveness of health voucher scheme and micro-health insurance scheme to support the poor and extreme poor in selected urban areas of Bangladesh: An assessment using a mixed-method approach
Source: PLoS One. 2021 Nov 1;16(11):e0256067. doi: 10.1371/journal.pone.0256067 (PMC8559931; doi:10.1371/journal.pone.0256067)
Supplement: S1 File — (PDF) [file pone.0256067.s007.pdf]

Focus Group Discussion guideline for beneficiaries

Protocol Title: Effectiveness of different financing options to support the extreme poor, including: voucher scheme and micro-health insurance

Investigator's name: Dr. Ziaul Islam
Organization: icddr,b

Questionnaire ID:

Guideline for Focused Group Discussion (FGD)
Themes of FGD
1. Knowledge of voucher system /micro-health insurance.
2. Experience of using voucher system /micro-health insurance
3. Satisfaction on the voucher system/ micro-health insurance
4. Barriers to utilize services under the scheme
5. Effectiveness of IEC strategies.
6. Opinion/ Suggestion on improvement

Talking points

১. ভাউচার কর্মসূচী/ মাইক্রোস্বাস্থ্যবীমা কর্মসূচী সম্পর্কে আপনারা কি জানেন? প্রোবঃ "ভাউচার কর্মসূচী/ মাইক্রোস্বাস্থ্যবীমা কর্মসূচী সম্পর্কিত জ্ঞান, কিভাবে কাজ করে, কিভাবে সেবা দেয়, কি কি সুযোগ সুবিধা পাওয়া যায় ইত্যাদি।
২. ভাউচার কর্মসূচী / মাইক্রোস্বাস্থ্যবীমা কর্মসূচী সম্পর্কে আপনিআপনারা কিভাবে জেনেছেন/? প্রোবঃ কে এটা সম্পর্কে বলেছে, কোন কোন মাধ্যমে জানতে পেরেছেন, কিভাবে সহজে জানতে পেরেছেন, কিভাবে জানানো হলে আরো সহজে জানতে পারতেন ?
৩. আপনারা কিভাবে ভাউচার কর্মসূচী/ মাইক্রোস্বাস্থ্যবীমা কর্মসূচী এর অন্তর্ভুক্ত হয়েছেন? প্রোবঃ কারা রেজিস্ট্রেশন করেছে , কিভাবে রেজিস্ট্রেশন করা হয়েছে ইত্যাদি।
৪. আপনাদের মাঝে বেশির ভাগ মানুষ অসুস্থ হলে সাধারণত কোথায় চিকিৎসা নেয়? প্রোবঃ কোন সেবা কেন্দ্রে যায়।
৫. স্বাস্থ্য কেন্দ্রে পৌছানোর পর আপনারা ভাউচার কর্মসূচী/মাইক্রোস্বাস্থ্যবীমা কর্মসূচীর কার্ডের মাধ্যমে কিভাবে সেবা পেয়ে থাকেন? প্রোবঃ কার্ড দেখানো, ভর্তি হওয়া, সেবা প্রদান শুরু ইত্যাদি।
৬. ভাউচার কর্মসূচী/ মাইক্রোস্বাস্থ্যবীমা   কর্মসূচী এর মাধ্যমে আপনারা কি কি সেবা পান? প্রোবঃ কি কি রোগের চিকিৎসা সেবা দেয়, কতটি রোগের সেবা দেয়, কোন কোন সেবা অন্তর্ভুক্ত, যেমনঃ ঔষুধ, ডায়গনিসিস, রেফারেল ইত্যাদি।
৭. বহির্বিভাগে ভাউচার কর্মসূচী/ মাইক্রোস্বাস্থ্য বীমা- কর্মসূচী থেকে ঔষুধ প্রদানের ব্যবস্থা চালু করার প্রয়োজন আছে কি? প্রোবঃ বহির্বিভাগে ঔষুধ প্রদানের কেন দরকার, কি কি রোগের জন্য বেশি দরকার ইত্যাদি।
৮. যারা (সেবাকেন্দ্রর বুথ, সেবাদানকারী নার্স, ডাক্তার এবং অন্যান্য কর্মচারী) সেবা প্রদান করেন তারা আপনাদের সাথে কেমন আচরণ করেন? প্রোবঃ ভর্তির সময় ভালভাবে কথা বলেন কি না, আপনার সমস্যা নিয়ে কথা বলেন কি না, আপনার সাথে হাসিমুখেআন্তরিকতার সাথে কথা বলে কি না/, যথাযথ সময় দেন কি না।
৯. ভাউচার কর্মসূচী/ মাইক্রোস্বাস্থ্যবীমা কর্মসূচীর সেবাদানকারী সেবাকেন্দ্রের এর পরিস্কার-পরিচ্ছন্নতা কেমন?
১০. সেবা দানকারীদের দক্ষতা কেমন বলে আপনাদের ধারণা?
১১. ভাউচার কর্মসূচী/ মাইক্রোস্বাস্থ্যবীমা কর্মসূচীর কার্ড সেবা নিতে গিয়ে কেমন সময় অপেক্ষা করতে হয়?
১২. অসুস্থ হওয়া সত্বেও ভাউচার কর্মসূচী/ মাইক্রোস্বাস্থ্যবীমা কর্মসূচীর কার্ড ব্যবহার করে সেবা না নেওয়ার কারন কি?
১৩. কার্ডের মাধ্যমে সেবা নিতে গিয়ে আপনারা কি কোন ধরনের অসুবিধার সম্মুখীন হয়েছিলেন? প্রোবঃ কি কি ধরনের অসুবিধা।
১৪. ভাউচার কর্মসূচী/ মাইক্রোস্বাস্থ্য বীমা- নিয়ে আপনার কোন মতামতপরামর্শ আছে/? প্রোবঃ কি করলে আরো ভাল হয়, আরো ভাল সেবা পাওয়া যায়, কিভেবে সঠিক লোক ভাউচার কর্মসূচী/ মাইক্রোস্বাস্থ্যবীমা কার্ড পাবে ইত্যাদি।
১৫. আর কোন কোন উদ্যোগ নিলে আরো বেশি মানুষ ভাউচার কর্মসূচী/ সম্পর্কে জানতে পারবে ?

Guideline for Key Informant Interview
(Managers/officials/service providers)

Protocol Title: Effectiveness of different financing options to support the extreme poor, including: voucher scheme and micro-health insurance

Protocol # PR-19084

Principal Investigator: Dr. Ziaul Islam
Organization: icddr, b

সাক্ষাৎকার গ্রহনকারীর জন্য নির্দেশনাঃ সাক্ষাৎকার গ্রহনের শুরুতে সম্মতিপত্র সম্পর্কে বোঝানো ও সাক্ষর করা নিশ্চিত করুন। সাক্ষাৎকার প্রদানকারীকে পুনরায় জিজ্ঞেস করুন যে এসব মন্তব্যের বাইরে তার আরও কিছু বলার আছে কি না। পরিশেষে তাকে ধন্যবাদ জানিয়ে সাক্ষাৎকার শেষ করুন।

সাক্ষাৎকার প্রদানকারীর সংশ্লিষ্টতা (সাধারণ তথ্য)

সাক্ষাৎকার গ্রহনের তারিখ:

সাক্ষাৎকার প্রদানকারীর নাম ও প্রতিষ্ঠানের নামঃ

সাক্ষাৎকার প্রদানকারীর থিকানাঃ

সাক্ষাৎকার গ্রহীতার নাম

Themes of KII (respondent’s engagement, experience, knowledge, perception, opinion and suggestions on the following issues

1. Role and responsibility of the respondent in the scheme/project
2. Information about common health problems of the beneficiaries
3. Strengths, weaknesses and opportunities of the scheme/ project
4. Gaps and challenges in service delivery (needs versus availability, infrastructure, HR, training, other supplies, BCC, patient satisfaction, community participation etc)
5. Potential for expansion of service package, and scaling up
6. Experience of payment mechanism for provider/ claim settlement process
7. Opinion on financial and programmatic sustainability of the scheme

Talking points

১. ভাউচার কর্মসূচী/ মাইক্রো স্বাস্থ্যবীমা কর্মসূচী সম্পর্কে বলুন?
২. এই কর্মসূচী কিভাবে পরিচালিত হয়?
৩. এই কর্মসূচীতে আপনার ভূমিকা ও সম্পৃক্ততা সম্পর্কে বলুন?
৪. এই কর্মসূচী নিয়ে আপনার মতামত কি?
৫. এই কর্মসূচীতে সাধারনত সুবিধাভোগিরা কি ধরনের স্বাস্থ্য সমস্যার সম্মুখিন হয়?
৬. এই কর্মসূচীতে অন্তভুক্ত সুবিধাভোগিরা কি ধরেনের সুবিধা পেয়ে থাকে?
৭. এই কর্মসূচীতে সুবিধাভোগিরা কি ধরনের স্বাস্থ্য সুবিধা পেয়ে থাকে?

৮. এই কর্মসূচীতে সুবিধাভোগিরা কি ধরনের সুবিধা দেওয়ার কথা? উল্লেখিত প্রাপ্তব্য সুবিধাগুলো কি সব পেয়ে থাকে? কি কি প্রাপ্তব্য সুবিধাগুলো এখনো প্রদান করা হয় না? উল্লেখিত প্রাপ্তব্য সুবিধাগুলো পেতে কি ধরনের পদক্ষেপ নেওয়া উচিত বলে মনে করেন?
৯. এই কর্মসূচী বাস্তবায়নের পথে চ্যালেঞ্জগুলো কি কি বলে আপনি মনে করেন?
১০. এই প্রকল্পের দায়িত্ব পালনকালে আপনি কি ধরনের চ্যালেঞ্জের সম্মুখীন হয়েছেন? কিভাবে এই চ্যালেঞ্জ সমূহ মোকাবেলা করেছেন?
১১. এই প্রকল্পের কাজ করতে গিয়ে আপনার পেশাগত অন্যান্য দায়িত্ব পালনে কি ধরনের ব্যঘাত ঘটে?
১২. এই প্রকল্পের সেবা প্যাকেজ সম্প্রসারণের জন্য কি ধরনের পদক্ষেপ নেয়া যায় ? এই সম্পর্কে আপনার মতামত কি?
১৩. এই প্রকল্পের প্রোভাইডার পেমেন্ট প্রক্রিয়া কিভাবে করা হয়? এই সম্পর্কে কি চ্যালেঞ্জের সম্মুখীন হতে হয়েছিল? উল্লেখিত চ্যালেঞ্জ সমূহ কিভাবে সমাধান করা যায়?
১৪. এই প্রকল্পটি টেকসই করার জন্য আরো কি কি পদক্ষেপ নেওয়া উচিত বলে আপনি মনে করেন?
